# The effect of auriculotherapy on individuals with anxiety in follow-up in Primary Health Care

**DOI:** 10.1590/0034-7167-2024-0186

**Published:** 2025-07-11

**Authors:** José Henrique da Silva Cunha, Vandilson Pinheiro Rodrigues, Heloísa Cristina Figueiredo Frizzo, Franciele Kavafara Pires, Amanda Polin Pereira, Francisca Bruna Arruda Aragão, Regina Célia Fiorati

**Affiliations:** I Universidade de São Paulo. Ribeirão Preto, São Paulo, Brazil; II Universidade do Maranhão. São Luís, Maranhão, Brazil; III Universidade Federal do Triângulo Mineiro. Uberaba, Minas Gerais, Brazil

**Keywords:** Auriculotherapy, Anxiety, Primary Health Care, Mental Health, Complementary Therapies, Auriculoterapia, Ansiedad, Atención Primaria de Salud, Salud Mental, Terapias Complementarias

## Abstract

**Objectives:**

to analyze the effects of auriculotherapy on the mental health of individuals with anxiety in Primary Healthcare.

**Methods:**

a sequential explanatory mixed-methods study was conducted with 60 patients from a health unit in Uberaba, MG. Quantitative data were obtained using the DASS-21 and WHOQOL-Bref questionnaires. Qualitative data were collected from 17 semi-structured interviews and analyzed through thematic content analysis. Statistical analysis included the Wilcoxon signed-rank test, paired t-test, and multivariate regression models.

**Results:**

after ten sessions of auriculotherapy, anxiety levels decreased, with most participants (86.7%) reaching normal levels. Additionally, 61.7% rated their quality of life as good, and 46.7% reported satisfaction with their health. Six thematic categories emerged. Auriculotherapy reduced DASS-21 scores and WHOQOL-Bref domain scores, even after adjusted analysis.

**Conclusions:**

auriculotherapy was effective in reducing anxiety and improving users’ quality of life.

## RESUMEN

**Objetivos:**

analizar los efectos de la auriculoterapia en la salud mental de individuos con ansiedad en la Atención Primaria de Salud.

**Métodos:**

se realizó un estudio mixto secuencial explicativo con 60 pacientes de una unidad de salud en Uberaba-MG. Los datos cuantitativos se obtuvieron mediante los cuestionarios DASS-21 y WHOQOL-Bref. Los datos cualitativos se recopilaron a partir de 17 entrevistas semiestructuradas y se analizaron mediante análisis de contenido temático. El análisis estadístico incluyó la prueba de rangos con signo de Wilcoxon, la prueba t para muestras dependientes y modelos de regresión multivariada.

**Resultados:**

después de diez sesiones de auriculoterapia, la ansiedad disminuyó, con la mayoría (86,7%) alcanzando niveles normales. Además, el 61,7% evaluó su calidad de vida como buena, y el 46,7% se mostró satisfecho con su salud. Emergieron seis categorías temáticas. La auriculoterapia redujo los puntajes del DASS-21 y los dominios del WHOQOL-Bref incluso después del análisis ajustado.

**Conclusiones:**

la auriculoterapia fue útil para reducir la ansiedad y mejorar la calidad de vida de los usuarios.

## INTRODUCTION

Auriculotherapy is a practice of Traditional Chinese Medicine (TCM) regulated as an Integrative and Complementary Health Practice (ICHP) in the Brazilian Healthcare system (In Portuguese, *Sistema Único de Saúde* - SUS). This type of therapy aims to stimulate points in the ear, regulating and balancing the body’s physiological performance^(1)^. Evidence has reinforced that auriculotherapy is highly acceptable to patients, safe and effective, with positive effects on physical, psychological and mental disorders, and can be used both preventively and therapeutically^(2,3)^.

This practice has a potential effect on reducing stress levels and improving the quality of life of healthcare professionals in the nursing team^(3)^. Auriculotherapy has been used to balance and unblock energy flows that cause illness, promoting the regulation and harmony of the body’s energetic functions and emotions^(4)^. A systematic review has also demonstrated the beneficial effect of auriculotherapy in reducing symptoms of stress, anxiety and depression in adults and older adults^(5)^.

It is important to note that anxiety is an important natural response for humans to respond to a certain danger^(6)^. In excess, it can become pathological, and has been considered one of the most frequent behavioral changes in the general population, and can negatively affect health and quality of life, in addition to being among the main causes of disability in the world^(7,8)^.

Studies have shown that there is little knowledge among Primary Healthcare (PHC) professionals and users regarding ICHP and their potential for application in care^(9,10)^. The lack of studies on the subject in PHC^(5)^ and the absence of qualified professionals in the SUS network mean that ICHP are included gradually and slowly^(10)^. Studies aimed at investigating the application of ICHP in PHC units in Brazil could assist professionals and managers in planning new actions, considering health teams’ local context and technical skills.

In this context, it is important to reinforce that PHC is recognized as a strategy for organizing the healthcare system, in the sense of guiding the distribution of resources to meet the population’s needs and coordinate the Healthcare Network, following principles such as first contact, longitudinality, comprehensiveness, coordination, family focus and community orientation^(11,12)^. Furthermore, PHC as a strategy will only exist if it fulfills three essential functions: resolution (resolving most health problems); communication (coordinating flows of people and information); and accountability (knowing and managing the population served)^(12)^.

Furthermore, the World Health Organization and the Brazilian National Policy on Integrative and Complementary Practices recommend the inclusion of integrative practices, such as auriculotherapy, in PHC. These practices are aligned with SUS principles, such as universality, accessibility, connection, continuity of care, comprehensiveness, accountability, humanization, equity and social participation^(13)^. The inclusion of these practices can expand users’ access to healthcare services previously restricted to private practices, benefiting the population more comprehensively^(14)^.

Thus, the present study is justified by the importance of analyzing the effects of auriculotherapy in promoting mental health in PHC users, contributing to knowledge production about the applicability of auriculotherapy in anxiety treatment, based on its action in promoting mental health, and subsidies for formulating public policies and action strategies, in search of comprehensive care. The present study has as its guiding question: what are the effects of auriculotherapy in promoting mental health in users with complaints of anxiety in the context of PHC?

## OBJECTIVES

To analyze the effects of auriculotherapy on the mental health of individuals with anxiety undergoing follow-up in PHC.

## METHODS

### Ethical aspects

This study was previously submitted and approved by the *Escola de Enfermagem de Ribeirão Preto* Research Ethics Committee. All participants were informed about the study stages and procedures and signed the Informed Consent Form.

### Study design, period and location

A mixed study of a sequential explanatory nature was conducted, with a first phase of quantitative analysis, followed by a second phase of qualitative analysis^(15)^. The first phase (quantitative) covered the period from June 2021 to February 2022, while the second phase (qualitative) covered the period from April to June 2022, following COnsolidated criteria for REporting Qualitative research guidelines.

This study was conducted at the *Unidade Matricial de Saúde Valdemar Hial Júnior*, located in the city of Uberaba, Minas Gerais, where approximately nine thousand monthly appointments are carried out, and has three Family Health Strategy (FHS) teams, which are Santa Terezinha, Jardim Espírito Santo and Fabrício.

### Population: inclusion and exclusion criteria

Participants of both sexes, aged 18 years or older, treated at the health unit with complaints of anxiety in the health unit’s care record, classified as having an anxiety level ranging from mild to extremely severe in the initial assessment of DASS-21^(16)^, and who were not undergoing another ICHP were included. Individuals who showed signs of infection, inflammation, or injury to the ear, used ear piercings (except earrings), were pregnant, were experiencing a psychotic episode or acute phase of any disease, did not attend two or more consecutive sessions, started drug treatment for anxiety, or were diagnosed with COVID-19 within a period of less than 15 days from the start of study treatment were excluded. It is noteworthy that no participant was excluded in the initial screening, since all users indicated by healthcare professionals met the established eligibility criteria.

For this study, a convenience sample was adopted. Initially, in the quantitative phase, the research team met with healthcare professionals from the three FHS teams (Santa Terezinha, Jardim Espírito Santo and Fabrício). During the meeting, therapeutic indications of auriculotherapy, its potential for the care of users and research objectives were discussed. Professionals were then asked to indicate users with anxiety. After the meeting, professionals initially provided a list of 31 users. Each of them was contacted by telephone and/or in person and invited to participate in the study. To be included, users had to present, in the initial DASS-21 assessment, a level of anxiety ranging from mild to extremely severe. This criterion was adopted to be able to assess the effect of auriculotherapy on the DASS-21 anxiety scores obtained before and after therapy.

To calculate sample size, we used the R statistical package version 4 (R Foundation for Statistical Computing, Vienna, Austria) resources. Calculation was based on the DASS-21 anxiety scores obtained before and after auriculotherapy in the sample of 31 participants initially recruited for the pilot study. Thus, adopting a difference of 5 points in DASS-21 anxiety scores, a significance level of 5% and a study power of 90%. The minimum sample size required was 60 participants.

The 31 participants from the pilot study were included in the final sample and, after further discussion with the team, they provided a new list of 29 potential participants. They were contacted and agreed to participate in the study, but two withdrew for personal reasons after completing seven and nine auriculotherapy sessions, respectively. To reach the sample of 60 participants, two new users were added, who also agreed to participate and were recommended by the unit healthcare professionals.

In the qualitative phase, participants were intentionally selected based on the results of the data collection instruments. This included participants who, after ten auriculotherapy sessions, presented normal levels of anxiety, perceived a good quality of life, and were satisfied with their health condition, and those who perceived a poor quality of life and dissatisfaction with their health condition. In this phase, 17 participants agreed to participate.

### Data collection: instruments and procedures

In the quantitative phase, participant socioeconomic characteristics were collected. Anxiety, stress and depression scores were assessed using the DASS-21^(16)^. The WHOQOL-Bref was used to analyze participants’ quality of life and satisfaction with their health. This instrument encompasses the following domains: physical, psychological, social relationships and environment^(17)^. It is worth noting that the application of auriculotherapy in the health unit studied was carried out from 8:00 a.m. to 6:00 p.m., and each participant had a scheduled time. At the end of the ten auriculotherapy sessions, DASS-21 and WHOQOL-Bref were reapplied and analyzed to assess participants’ anxiety, quality of life and satisfaction with their health before and after the application of auriculotherapy.

The qualitative phase consisted of semi-structured interviews, developed and refined by the researchers, participant observation^(18)^ and field diary^(19)^, containing records of participants’ perceptions about the auriculotherapy sessions and the researcher’s observations of the observed reality. In this stage, participants were contacted by telephone, and interviews were recorded on cell phones, taking place individually in a private location of the Basic Health Unit. With a mean duration of one hour, times were scheduled according to participants’ availability. Transcripts were double-checked by the main researcher to ensure integrity, and were subsequently validated by participants via WhatsApp^®^.

Each participant participated in ten weekly auriculotherapy sessions, each lasting 30 minutes. During these sessions, specific points, such as *Shenmen*, kidney, sympathetic nervous system, liver, muscle relaxation, brainstem, heart, lung, and anxiety 1 and 2, were stimulated using disposable and sterile 0.18 × 8 mm stainless steel needles. After the sessions, the needles were replaced with cowpea seeds fixed with anti-allergenic microporous tape to the same auricular points. The auricular points were rotated, and participants were instructed to stimulate the cowpea seeds three times a day with moderate finger pressure, and to remove them after seven days to avoid saturation of the nerve receptors and possible pressure injuries. The definition of the points, their location, and the number of sessions followed evidence for anxiety^(5,20)^. The main researcher of this study, specialized in acupuncture, collected quantitative and qualitative data, and followed biosafety standards to apply auriculotherapy (use of latex gloves, cotton and 70% ethyl alcohol to clean the ear).

### Statistical analysis

Data analysis was performed using the R statistical package version 4 (R Foundation for Statistical Computing, Vienna, Austria). Descriptive analysis included summary measures of absolute and relative frequency, mean and standard deviation. Comparative analysis of the distribution of ordinal categories between time points (before and after) was conducted using the Wilcoxon signed-rank test. The Shapiro-Wilk test was used to verify the assumption of normality in the distribution of continuous variables obtained from the DASS-21 scores and the WHOQOL-Bref domains. After this processing, Student’s t-test for dependent samples was applied to analyze the differences in scores between time points.

Moreover, multivariate linear regression models were used to assess the effect of the intervention on the DASS-21 scores and WHOQOL-Bref domains, adjusted for the factors of age, sex, marital status, education, and religion. This multivariate analysis aimed to reduce confounding bias in the analysis of the effect of the intervention and to identify other factors associated with changes in the scores. The Akaike Information Criterion^(21)^ was used to select the dependent variable among the possible models of counting the GAMLSS class, in the case of the DASS-21, and among the set of real ones, in the case of the WHOQOL-Bref. To assess the adjusted regression model adequacy, the Shapiro-Wilk normality test was applied to adjustment residuals. The significance level adopted for all tests was 5%.

### Qualitative analysis

This process was based on a cyclical triad, involving the theoretical framework^(12)^, semi-structured interviews and participant observation, and the use of ATLAS.ti version 22, which assisted in the process of organizing, coding and optimizing the analytical process of qualitative data^(22)^. During thematic content analysis^(18)^, the following phases were followed: pre-analysis, material exploration, treatment and interpretation of the results obtained. During pre-analysis, the material organization and skimming from the interviews and participant observation took place, which were transcribed and inserted into ATLAS.ti. Each interview was coded by sequential number of participants (P1, P2...). In material exploration, the themes were grouped according to their content, based on the units of meaning, until the categories were formed. The units of participants’ speeches were selected/cut (called quotations in the program), and codes were created and assigned, according to repetitions of similar content present in their speeches (quotations), which were grouped into categories (families). In interpretation of results, articulations were made between quotations, codes and categories, generating visualization networks, in the form of thematic categories, discussed based on the theoretical framework on “PHC as a strategy for organizing the healthcare system”^(12)^.

## RESULTS

### Quantitative phase results

A total of 60 participants, 88.3% women and 11.7% men, with a mean age of 38.3 years (range 18 to 66 years), were included in this study. The majority reported being single (50%), with 48.3% having completed higher education. Among the participants with higher education, the majority had a higher education degree, but did not work in the field of training and worked in another profession, such as administrative assistant (n = 06) and community health worker (n = 3), and one of the community workers was also a physical education professional, makeup artist (n = 1), self-employed (n = 2), dental surgeon’s assistant (n = 1), sales representative (n = 1) and houseperson (n = 1). There were also other professionals who were working in their field of training and who lived in the territory, such as nurses (n=6), dentists (n=2), nutritionists (n=1), biomedical scientists (n=1), veterinarians (n=1), lawyers (n=2) and social workers (n=1). It was observed that 35% reported not belonging to any religion. Regarding their profession, most participants were administrative assistants (13.3%).

There was a reduction in the percentage of the most affected categories of anxiety, stress and depression in the sample after treatment with auriculotherapy (p < 0.001). There was also a statistically significant improvement in the classification pattern of self-perception of quality of life and satisfaction with health condition (p < 0.001). It is noteworthy that, before treatment, the majority of participants (45%) assessed their quality of life as neither good nor bad, and 35% were neither satisfied nor dissatisfied with their health. After treatment, 61.7% of participants began to assess their quality of life as good, and 46.7% were satisfied with their health (Table 1).

**Table 1:** Classification of anxiety, stress, depression and quality of life levels (WHOQOL-Bref) of participants before and after auriculotherapy treatment (N=60), Uberaba, Minas Gerais, Brazil, 2023

Variables	Before		After		*p* value
	n	(%)	n	(%)	
Anxiety					<0.001
Normal	0	(0.0%)	52	(86.7%)	
Mild	5	(8.3%)	3	(5.0%)	
Moderate	16	(26.7%)	5	(8.3%)	
Severe	2	(3.3%)	0	(0.0%)	
Extremely severe	37	(61.7%)	0	(0.0%)	
Stress					<0.001
Normal	2	(3.3%)	53	(88.3%)	
Mild	3	(5.0%)	5	(8.3%)	
Moderate	18	(30.0%)	2	(3.3%)	
Severe	22	(36.7%)	0	(0.0%)	
Extremely severe	15	(25.0%)	0	(0.0%)	
Depression					<0.001
Normal	11	(18.3%)	54	(90.0%)	
Mild	9	(15.0%)	2	(3.3%)	
Moderate	14	(23.3%)	4	(6.7%)	
Severe	12	(20.0%)	0	(0.0%)	
Extremely severe	14	(23.3%)	0	(0.0%)	
Self-perceived quality of life					<0.001
Very bad	1	(1.7)	0	(0.0)	
Bad	7	(11.7)	2	(3.3)	
Neither bad nor good	27	(45.0)	11	(18.3)	
Good	19	(31.7)	37	(61.7)	
Very good	6	(10.0)	10	(16.7)	
Health satisfaction					<0.001
Very dissatisfied	4	(6.7)	0	(0.0)	
Dissatisfied	17	(28.3)	7	(11.7)	
Neither satisfied nor dissatisfied	21	(35.0)	16	(26.7)	
Satisfied	15	(25.0)	28	(46.7)	
Very satisfied	3	(5.0)	9	(15.0)	

*Note: p-value was calculated using the Wilcoxon signed-rank test.*

There were statistically significant reductions after the intervention for the values of depression, anxiety and stress scores observed in the DASS-21 (p <0.001). The mean anxiety level score was 22.33 ± 9.49 (before) and 2.90 ± 3.30 (after). Concerning stress, before the mean score was 28.07 ± 7.30, and reduced to 8.50 ± 5.43. In the depression score, the mean observed was 18.93 ± 10.81 (before) and 3.67 ± 4.02 (after). In participants’ quality of life domains, there was a statistically significant increase (p <0.001) in the mean scores in all WHOQOL-Bref domains. After treatment, the physical domain presented a higher mean score (59.67 ± 12.20), followed by the social relationships (57.67 ± 11.95), psychological (55.44 ± 9.99) and environmental (54.88 ± 11.77) domains, shown in Table 2.

**Table 2:** Summary measures of DASS-21 scores and WHOQOL-Bref domains of participants before and after auriculotherapy treatment (N=60), Uberaba, Minas Gerais, Brazil, 2023

Variables	Before		After		*p* value
	Mean	Standard deviation	Mean	Standard deviation	
DASS-21 scores					
Depression	18.93	10.81	3.67	4.02	<0.001
Anxiety	22.33	9.49	2.90	3.30	<0.001
Stress	28.07	7.30	8.50	5.42	<0.001
WHOQOL-Bref domains					
Physical	41.29	13.25	59.67	12.20	<0.001
Psychological	40.11	11.68	55.44	9.99	<0.001
Social relationships	44.33	14.97	57.67	11.95	<0.001
Environment	46.79	11.46	54.88	11.77	<0.001

*Note: p-value was calculated using Student’s t-test for dependent samples.*

Table 3 shows the multivariate regression model analysis of the effect of the intervention on anxiety, stress and depression scores, adjusted for age, sex, education, marital status and religion. The data indicate a mean relative reduction of 87.72% (Estimate = -2.097; p <0.001) in the anxiety domain. The higher level of education led to a mean relative reduction of 22.91% (Estimate = -0.260; p = 0.019) in the anxiety domain. The intervention showed a significant relative reduction of 69.46% (Estimate = - 1.186; p <0.001) in the mean score of the stress domain. There was a mean relative reduction of 82.51% (Estimate = -1.743; p <0.001) in the mean depression score. In participants with higher education, there was a mean relative reduction of 37.02% (Estimate = -0.462; p = 0.001) in the mean depression score. Regarding the WHOQOL-Bref quality of life domains, they were adjusted for sociodemographic factors.

**Table 3:** Multivariate regression model of the effect of the intervention on DASS-21 and WHOQOL-Bref domains adjusted for sociodemographic factors, Uberaba, Minas Gerais, Brazil, 2023

Dependent variables	Factors	Adjusted estimate	Standard error	t	*p* value
DASS-21 scores					
Anxiety	Intercept	2.355	0.282	8.336	0.000
	**Intervention**	-2.097	0.167	12.499	0.000
	Age	0.002	0.005	0.416	0.678
	Male	-0.227	0.177	-1.283	0.201
	Marital status (single)	0.088	0.116	0.755	0.451
	Education	-0.260	0.110	-2.365	0.019
	Spiritist religion	0.068	0.136	0.498	0.619
	No religion	0.130	0.137	0.948	0.344
	Other religion	0.037	0.201	0.185	0.853
Stress	Intercept	2.696	0.172	15.637	0.000
	**Intervention**	-1.186	0.091	-2.918	0.000
	Age	-0.001	0.003	-0.605	0.546
	Male	-0.116	0.102	-1.140	0.256
	Marital status (single)	0.018	0.070	0.267	0.789
	Education	-0.096	0.065	-1.467	0.145
	Spiritist religion	0.034	0.084	0.411	0.681
	No religion	0.134	0.082	1.628	0.106
	Other religion	0.060	0.117	0.510	0.610
Depression	Intercept	2.634	0.344	7.651	0.000
	**Intervention**	-1.743	0.187	-9.324	0.000
	Age	-0.004	0.006	-0.679	0.498
	Male	-0.449	0.238	-1.883	0.062
	Marital status (single)	0.115	0.148	0.774	0.440
	Education	-0.462	0.138	-3.348	0.001
	Spiritist religion	-0.149	0.170	-0.877	0.382
	No religion	0.018	0.166	0.110	0.912
	Other religion	-0.118	0.260	-0.454	0.650
WHOQOL-Bref					
Physical	Intercept	3.343	0.210	15.902	0.000
	**Intervention**	0.981	0.094	10.433	0.000
	Age	-0.011	0.005	-2.023	0.045
	Male	0.353	0.161	2.193	0.030
	Marital status (single)	0.060	0.098	0.615	0.539
	Education	0.325	0.101	3.217	0.001
	Spiritist religion	0.079	0.189	0.417	0.676
	No religion	-0.306	0.113	-2.712	0.007
	Other religion	-0.198	0.288	-0.686	0.493
Environment	Intercept	3.678	0.340	10.795	0.000
	**Intervention**	0.387	0.098	3.958	0.000
	Age	-0.014	0.005	-2.752	0.006
	Male	0.242	0.144	1.678	0.096
	Marital status (single)	0.197	0.110	1.790	0.076
	Education	0.141	0.096	1.471	0.144
	Spiritist religion	0.180	0.172	1.044	0.298
	No religion	-0.044	0.188	-0.234	0.814
	Other religion	-0.076	0.217	-0.353	0.724
Psychological	Intercept	3.080	0.251	12.245	0.000
	**Intervention**	0.766	0.088	8.707	0.000
	Age	-0.003	0.004	-0.689	0.491
	Male	0.339	0.145	2.338	0.021
	Marital status (single)	0.009	0.101	0.096	0.923
	Education	0.200	0.096	2.086	0.039
	Spiritist religion	0.125	0.120	1.038	0.301
	No religion	-0.328	0.120	-2.733	0.007
	Other religion	-0.192	0.168	-1.142	0.256
Social relationships	Intercept	1.303	0.077	16.91	0.000
	**Intervention**	0.186	0.032	5.699	0.000
	Age	-0.002	0.001	-2.227	0.027
	Male	0.087	0.040	2.155	0.033
	Marital status (single)	0.007	0.026	0.291	0.771
	Education	-0.055	0.028	-1.960	0.052
	Spiritist religion	0.034	0.034	0.979	0.329
	No religion	-0.061	0.038	-1.615	0.109
	Other religion	0.007	0.048	0.155	0.876

The main result of quantitative analysis was that the auriculotherapy intervention had an effect on improving all quality of life domains. In addition, older age was related to lower scores in the physical, environmental and social relationships domains. Male sex was related to higher scores in the physical, psychological and social relationships domains. Higher education was related to higher scores in the physical and psychological domains. And the category without religion was related to lower scores in the physical and psychological domains.

### Qualitative phase results

In the qualitative phase, 17 participants agreed to participate. Most of them (n=14) were female, with a mean age of 40.58 years (26-58 years). Furthermore, 11 were married and six were single, with the majority (n=8) having completed higher education, with a predominance of non-religious individuals (n=8) and community health workers (n=3). From the thematic content analysis of qualitative data with the support of ATLAS.ti version 22, six categories emerged (Chart 1).

**Chart 1:** Organization of data using codes and categories (families), according to the use of ATLAS.ti version 22, Uberaba, Minas Gerais, Brazil, 2023

Categories (families)	Codes
Limited knowledge of users regarding auriculotherapy and its potential for care	Knowledge of auriculotherapy; lack of knowledge of auriculotherapy; little knowledge of auriculotherapy.
Impact of anxiety on physical and mental health	Agitation; aggressiveness; skin allergy; nausea; increased blood pressure; increased desire to eat; increased desire to smoke; tiredness; conflicts with people close to them; difficulty sleeping; headache; nausea; memory loss; impatience; irritability; nervousness; racing thoughts; negative thoughts (death and fear of something bad happening); worry; hair loss; tachycardia.
Repercussions of the pandemic on psychological, economic, social and physical factors	Anxiety; increased anxiety due to working in a healthcare environment; coping with the loss of loved ones; financial difficulties; difficulty sleeping; social distancing; stress; impact on occupation/work; fear of dying; fear of losing loved ones; fear of being infected by the coronavirus; nervousness; violence against women.
Benefits of auriculotherapy for health promotion and lifestyle change	Self-knowledge and calmness to deal with adverse situations; contributes to quality of life; contributes to satisfaction with health; co-responsibility in healthcare; balance of blood pressure; maintenance of health; improvement of headaches; improvement of body pain; improvement of premenstrual tension (PMS); improvement of nervousness and fear; improvement of family relationships; improvement of sleep; improvement and control of anxiety; promotes awakening to a healthy lifestyle; promotes time to take care of oneself; reduction of medication dosage for anxiety; reduction of hair loss.
Recognition of auriculotherapy as a significant healthcare resource	Auriculotherapy as a natural resource in healthcare; the effect of auriculotherapy is long-lasting; refusal to take allopathic medicine (dependence and side effects).
Negative factors correlated with mental health, quality of life and health satisfaction	Family conflict; disorganized routine; financial difficulties; pain; migraine; unhealthy lifestyle; multiple role-playing; Fibromyalgia; death of a loved one; abusive relationship.

In category 1, there was a lack of knowledge about auriculotherapy, its provision and potential for care in PHC, as per the statements:

*I had already heard about it. But I didn’t know anything about it.* (P5)

*I had, like, very superficial. My mother did this treatment, she had back problems, some physical problems, right?* (P39)

In category 2, the impact of anxiety on physical and mental health were aspects with the highest number of codes in ATLAS.ti. Physical symptoms included headache, hair loss, fatigue, skin allergy, tachycardia, increased blood pressure, nausea and vomiting:

*I had so many headaches. It was a migraine, it was nausea* [...] *my blood pressure was very high* [...] *my hair loss was absurd, very strong.* (P57)

[...] *I also had hair loss and I thought I was going to go bald* [...] *I was sometimes having tachycardia, you know? In moments of crisis* [anxiety]. (P2)

[...] *an allergy* [on the skin] *from anxiety before the treatment* [...] *a lot of anxiety, then I would sometimes start to feel nauseous.* (P41)

Anxiety caused accelerated thinking, memory lapses, negative thoughts, worry, agitation, impatience, irritability, nervousness and difficulty sleeping:

[...] *I was feeling extremely tired, but with agitation and very accelerated thinking and I didn’t sleep well at night* [...]*. And so, I started having problems, with memory lapses, slow reasoning* [...]. (P21)

*I had a very accelerated mind* [...] *my anxiety was generalized, thinking that I was going to die at any moment* [...]. (P55)

*I didn’t sleep, I didn’t rest, I woke up nervous, I woke up feeling rushed* [...]. (P41)

As a result of anxiety, some participants reported turning to food:

[...] *I was harming my body because I took out this anxiety on food, on sweets* [...] *I felt anxious, I ate.* (P54)

[...] *I didn’t sleep well; I took out my anxiety by eating at night.* (P57)

Anxiety increased the desire to smoke and caused conflicts with people close to them:

*I couldn’t coordinate or organize my thoughts, and this ended up destabilizing me even more, making me more anxious, smoking a lot, taking it out on myself, fighting with people close to me.* (P17)

In category 3, it was observed that the COVID-19 pandemic generated anxiety, worry, fear and death of loved ones:

*My anxiety increased* [...] *I lost two of my neighbors, one upstairs and the other downstairs. I lost two cousins* [...] *and then we started to feel desperate and I became a bit desperate* [...] *I was desperate because of my mother too, because she was an older adults. I was desperate because I had high blood pressure. We couldn’t catch COVID.* (P57)

*I started having anxiety attacks* [...] *I was very afraid of catching COVID, very afraid of dying. Very afraid of passing it on to my family* [...] *I spent a long time without going to their house* [...]. (nurse/P21)

*I started to have this anxiety at the very beginning of the pandemic* [...] *I think it’s because of my work, because I’m a healthcare professional, right? We work on the front line.* (community worker/P6)

Furthermore, the pandemic caused social distancing, financial difficulties, and there were cases of violence against women:

*And also, the isolation, right? We had to, it was work and home, right? You didn’t have a social life anymore.* (P6)

*In the financial aspect, because as I work with events, I found myself in a position where I could no longer work* [...] *this really, really affected my economic and psychological life* [...]. (P24)

*At the beginning of the pandemic, I went through a huge trauma and that made me develop. The anxiety I already had got worse* [...] *he* [ex-partner] *tried to take my life* [...] *I managed to escape* [...]. (P54)

In category 4, benefits of auriculotherapy were observed for health promotion, lifestyle changes and family relationships:

*I noticed that my anxiety improved a lot* [...] *it improved, I can tell you that it improved 100%* [...] *it really improved a lot, because I felt much more in control and relaxed.* (P5)

*The treatment* [auriculotherapy] *slowed me down* [...] *now it* [anxiety] *is stable, right? I can control it* [...]. (P41)

*It helped a lot, because it’s a pandemic and we’re in danger. Even family problems* [...] *completely change your attitude and you become calmer.* (P29)

*Wow, I think I’m a different person. I’m really good* [...] *much calmer, at home with my husband, with my children and with my work.* (P57)

The treatment helped participants take responsibility for their health and lead a healthy lifestyle:

*There was a person without doing an activity, without anything. Now at the moment, I am doing an activity; it has changed a lot.* (P5)

*I lost five kilos because I started to control my anxiety more, I reduced the amount of food, the things I was eating, the junk food.* (P17)

[...] *I started exercising again, so I completely changed my diet, I’m not eating sugar* [...] *I’m opting more for fruit and vegetables.* (P41)

In category 5, some participants expressed concern about the side effects of allopathic medicines and recognized auriculotherapy as a non-pharmacological therapeutic resource that can contribute to their physical and mental balance as well as to promoting their health:

*I went to a psychiatrist. He had prescribed strong medication for me, but I chose not to take it. I took it for three days and gave up* [...] *I thought it would work* [auriculotherapy treatment]*, but it didn’t work out that way. I expected a much smaller result and it went beyond what I imagined.* (P29)

*I didn’t want to take medicine* [...] *I think that the treatment* [auriculotherapy] *doesn’t just treat the mind, it treats, like I told you, the mind, it treats everything like that. I think it’s better than medicine, because medicine is temporary.* (P57)

In category 6, it was observed that some participants had experienced losing family members, causing harm to their physical and mental health as well as to their quality of life and satisfaction with their health:

[...] *but it was equalized by the sudden loss of my family* [...] *this loss was a heart attack that my uncle had, right. He had a heart attack and I felt sick, my blood pressure went up and I was scared about it and after that I was afraid, thinking it would happen to me* [...]. (P55)

*It started with the loss of my father* [...] *after he passed away, I felt a lot of pain in my spine. I thought I had a herniated disc. I went to the orthopedist* [...] *he said, really, you have fibromyalgia.* (P24)

Some participants did not observe any improvement in their quality of life or satisfaction with their health. The reasons for this included multiple roles, financial difficulties and family conflict:

[...] *I had to change some things that I was, like, doing or allowing others to do to me and that I needed to make decisions in this sense, learn to say no, and give priority, prioritize myself in relation to other people* [...] *learn to divide the tasks and not overload myself, for example, at home, right?* [...]. (P47)

[...] *financial. Family, everything bad. One follows the other, right? Even if we don’t want it to. One thing follows the other and one thing going wrong, when one thing goes wrong it’s already annoying, now imagine when everything goes wrong, it’s financial, it’s family.* (P4)

[...] *I have a lot of problems at home, specifically with my son. So, I can’t get through a day well.* (P24)

## DISCUSSION

The results of this study suggest that auriculotherapy may contribute to reducing anxiety levels. These findings are corroborated by other studies^(1,23)^ that found positive effects of auriculotherapy on anxiety. Most participants with anxiety were female. This finding is in line with a previous study^(24)^, which indicates that women may be more affected by anxiety due to several factors, such as greater social pressure and lower income compared to men, and many of these women adopt longer working hours to maintain their family’s economic conditions.

In addition to reducing anxiety, an improvement in symptoms of stress and depression was also observed, in line with the results of a systematic review^(5)^, which reinforced the effectiveness of auriculotherapy in reducing these symptoms. Another study^(25)^ also indicates that auriculotherapy may be useful in caring for people with depression.

Regarding participants’ level of education, most had completed higher education. This data contrasts with this study^(26)^, which revealed that a large proportion of primary care users had incomplete elementary education. This phenomenon can be explained by the fact that there were reports of participants who were facing economic difficulties and anxiety due to the COVID-19 pandemic, which may have led to an increase in individuals with higher education seeking treatment at the health unit in question. Data collection during the pandemic period may have contributed to the change in the profile of the public served by the health unit. Furthermore, many participants with higher education degrees were working in areas other than their original training, such as administrative assistant, community worker, makeup artist, self-employed, sales representative, dental surgeon assistant, and houseperson.

It is important to highlight that this study began during the COVID-19 pandemic, which may have negatively impacted participants’ mental health, generating anxiety, fear of contagion and death, and affecting social interaction and work. Evidence has shown that, in addition to triggering an economic and health crisis, the COVID-19 pandemic led the Brazilian State to adopt social isolation as a preventive measure^(27,28)^. However, a worrying side effect of social isolation has been the increase in domestic violence against women, as victims spend more time in contact with their aggressor, often their intimate partner^(28)^.

In relation to the improvement in quality of life and satisfaction with health, participants indicated that auriculotherapy enabled improvements in anxiety, sleep, headaches, reduced hair loss and balanced blood pressure. Furthermore, many began to take an active role in promoting their health, adopting a healthy lifestyle that includes regular physical activity and a healthy diet. These findings support data from previous studies_(29,30)_ that indicate that ICHP can increase primary care users’ awareness of their lives and the importance of self-care, in addition to contributing to reducing the use of medication and promoting protagonism and empowerment.

It is observed that the incorporation of ICHP in primary care can contribute not only to the establishment of a quality SUS, but also to solving users’ health problems. However, according to these studies^(5,31)^, ICHP are mainly performed in tertiary and secondary settings, which reinforces the need for more studies like this one in the scope of primary care, in order to strengthen the recognition of these practices, such as auriculotherapy, as a public health strategy in Brazil.

Therefore, it is crucial that there be, within the scope of primary care, ongoing education, to train health teams to use integrative practices and to disseminate the Brazilian National Policy of Integrative and Complementary Practices to healthcare professionals and primary care users, since many of these individuals are unaware of this policy and the potential of using these non-pharmacological practices for comprehensive healthcare^(30)^.

It is noteworthy that, in this study, it was found that auriculotherapy had a positive impact on reducing anxiety symptoms, resulting in an improvement in participants’ quality of life and health satisfaction. However, some faced economic and social challenges, such as financial difficulties, overload of responsibilities and family conflicts, which negatively affected their quality of life and health satisfaction. Therefore, it is crucial to adopt an interdisciplinary and intersectoral approach, involving different knowledge and sectors of society, to offer support to those who continue to experience an unsatisfactory quality of life and health satisfaction, even after treatment with auriculotherapy. From this perspective, intersectorality is an effective strategy to address health problems aggravated by social determinants of the health-disease process^(32)^, and the health sector, especially PHC, must work with other sectors and agents to ensure that public policies are aligned in order to maximize their potential to contribute to health and human development^(12)^.

One of the main contributions of this study is the use of the explanatory mixed method, which allowed obtaining quantitative results and their detailed explanation through interviews with participants in the qualitative phase, making it possible to assess, in a quantitative and qualitative manner, the effects of auriculotherapy on anxiety, quality of life and satisfaction with health. The results may be of great importance for health sciences, especially for ICHP. The results of this research demonstrated that auriculotherapy is a useful therapy to reduce anxiety, improve quality of life and satisfaction with health as well as to contribute to the promotion of mental health and comprehensive care. This discovery may have a significant contribution to the scientific community, which is dedicated to studies on auriculotherapy, as well as to the formulation of programs, strategies and health policies within the scope of PHC, expanding the therapeutic possibilities to increase the comprehensiveness of healthcare and, thus, strengthen the SUS.

### Study limitation

One of the limitations was that a single closed protocol of auriculotherapy points was used. Therefore, we suggest the development of future research with individualized points, following the precepts of TCM, based on energy imbalance, health condition and specific needs in a non-protocol and individualized way that guarantees better potential. However, the use of protocols allowed evidence and the dissemination of therapeutic practice through this study. It is also important to consider the possible placebo effect in studies that assess therapeutic effects. Therefore, we suggest future comparative studies that include other types of therapeutic groups, with or without the use of medications, with the aim of investigating the effects of auriculotherapy in comparison to other types of intervention. Another limitation was that this study was carried out during the pandemic, which may have reduced access and the sample of participants, which affects the generalizability of the study. We suggest the development of new longitudinal studies with longer follow-up to analyze the effects in the medium and long term.

### Contributions to nursing

Nursing plays a fundamental role in PHC users’ quality of life, and currently stands out in the scenario of use of ICHP for assistance and research within the vitalist paradigm and comprehensive care. In this regard, it is also relevant to emphasize the importance of interdisciplinary and intersectoral action, aiming to achieve a comprehensive and holistic approach to healthcare.

## CONCLUSIONS

The findings of this study suggest that auriculotherapy may be useful in reducing levels of anxiety, stress, and depression, and improving aspects related to participants’ quality of life and health satisfaction. Its inclusion in PHC may contribute to strengthening a comprehensive healthcare system that encompasses prevention, promotion, and recovery of health, with continuous, humanized, and comprehensive care. It is important that health managers train PHC professionals to use auriculotherapy as part of care and to publicize its benefits.

Further studies on auriculotherapy for other clinical conditions of PHC are recommended in order to disseminate and recognize this therapeutic approach. Furthermore, it would be beneficial to conduct comparative studies between the selection of individualized points based on TCM and the points used in this study, for a more detailed analysis of the effects of auriculotherapy on anxiety, as well as to conduct longitudinal studies to assess the efficacy of auriculotherapy.
