# Supplementary material for: The effect of auriculotherapy on individuals with anxiety in follow-up in Primary Health Care
Source: Rev Bras Enferm. 2025 Jul 11;78(3):e20240186. doi: 10.1590/0034-7167-2024-0186 (PMC12252924; doi:10.1590/0034-7167-2024-0186)
Supplement: Translation

# Translation: O efeito da auriculoterapia em indivíduos com ansiedade em seguimento na Atenção Primária à Saúde

**DOI:** 10.1590/0034-7167-2024-0186pt

**Authors:** José Henrique da Silva Cunha, Vandilson Pinheiro Rodrigues, Heloísa Cristina Figueiredo Frizzo, Franciele Kavafara Pires, Amanda Polin Pereira, Francisca Bruna Arruda Aragão, Regina Célia Fiorati

**Affiliations:** I Universidade de São Paulo. Ribeirão Preto, São Paulo, Brasil; II Universidade do Maranhão. São Luís, Maranhão, Brasil; III Universidade Federal do Triângulo Mineiro. Uberaba, Minas Gerais, Brasil

**Keywords:** Auriculoterapia, Ansiedade, Atenção Primária à, Saúde, Saúde Mental, Terapias Complementares

## Abstract

**Objetivos:**

analisar os efeitos da auriculoterapia na saúde mental de indivíduos com ansiedade na Atenção Primária à Saúde.

**Métodos:**

estudo misto sequencial explanatório, realizado com 60 pacientes de uma unidade de saúde em Uberaba-MG. Dados quantitativos foram obtidos pelos questionários DASS-21 e WHOQOL-Bref. Os dados qualitativos foram obtidos de 17 entrevistas semiestruturadas e analisados pela análise de conteúdo temática. A análise estatística incluiu o teste de postos sinalizados de Wilcoxon, o teste t para amostras dependentes e modelos de regressão multivariado.

**Resultados:**

após dez sessões de auriculoterapia, a ansiedade diminuiu, com a maioria (86,7%) atingindo níveis normais. Ademais, 61,7% avaliaram sua qualidade de vida como boa, e 46,7% ficaram satisfeitos com sua saúde. Emergiram seis categorias temáticas. A auriculoterapia reduziu os escores do DASS-21 e domínios do WHOQOL-Bref mesmo após análise ajustada.

**Conclusões:**

a auriculoterapia foi útil para reduzir a ansiedade e melhorar a qualidade de vida dos usuários.

## INTRODUÇÃO

A auriculoterapia é uma prática da Medicina Tradicional Chinesa (MTC) regulamentada como Prática Integrativa e Complementar em Saúde (PICS) no Sistema Único de Saúde (SUS). Esse tipo de terapia objetiva estimular pontos do pavilhão auricular, regulando e equilibrando o desempenho fisiológico do corpo^(1)^. Evidências têm reforçado que a auriculoterapia possui grande aceitabilidade pelos pacientes, segurança e eficácia, com efeitos positivos sobre distúrbios físicos, psíquicos e mentais, podendo ser utilizada tanto de forma preventiva quanto terapêutica^(2,3)^.

Essa prática possui efeito potencial na redução dos níveis de estresse e melhoria na qualidade de vida dos profissionais de saúde em equipe de enfermagem^(3)^. A auriculoterapia tem sido utilizada para equilibrar e desbloquear fluxos de energia que causam o adoecimento, promovendo a regulação e harmonia das funções energéticas do corpo e das emoções^(4)^. Revisão sistemática também tem demonstrado o efeito benéfico da auriculoterapia na redução de sintomas de estresse, ansiedade e depressão em indivíduos adultos e idosos^(5)^.

Destaca-se que a ansiedade é uma resposta natural importante para o ser humano responder a um determinado perigo^(6)^. Em excesso, pode se tornar patológica, e tem sido considerada uma das alterações comportamentais mais frequentes na população em geral, podendo afetar negativamente a saúde e a qualidade de vida, além de estar entre as principais causas de incapacidade no mundo^(7,8)^.

Estudos têm apontado que há pouco conhecimento entre profissionais e usuários da Atenção Primária à Saúde (APS) a respeito das PICS e suas potencialidades de aplicação no cuidado^(9,10)^. A escassez de estudo sobre a temática na APS^(5)^ e ausência de profissionais qualificados na rede do SUS fazem com que as PICS sejam incluídas de forma gradual e lentificada^(10)^. Estudos endereçados para a investigação da aplicação de PICS em unidades de APS no Brasil poderiam auxiliar profissionais e gestores no planejamento de novas ações, considerando o contexto local e as habilidades técnicas das equipes de saúde.

Nesse contexto, é importante reforçar que a APS é reconhecida como uma estratégia de organização do sistema de saúde, no sentido de orientar a distribuição de recursos para atender às necessidades da população e coordenar a Rede de Atenção à Saúde, seguindo princípios como primeiro contato, longitudinalidade, integralidade, coordenação, focalização na família e orientação comunitária^(11,12)^. Ademais, a APS como estratégia apenas existirá se cumprir três funções essenciais: resolubilidade (resolver a maioria dos problemas de saúde); comunicação (coordenar fluxos de pessoas e informações); e responsabilização (conhecer e gerenciar a população atendida)^(12)^.

Além disso, a Organização Mundial da Saúde e a Política Nacional de Práticas Integrativas e Complementares recomendam a inclusão de práticas integrativas, como a auriculoterapia, na APS. Essas práticas estão alinhadas com princípios do SUS, como universalidade, acessibilidade, vínculo, continuidade do cuidado, integralidade, responsabilização, humanização, equidade e participação social^(13)^. A inserção dessas práticas pode ampliar o acesso dos usuários a serviços de saúde anteriormente restritos a práticas de cunho privado, beneficiando a população de forma mais abrangente^(14)^.

Dessa forma, o presente estudo se justifica pela importância da análise dos efeitos da auriculoterapia na promoção da saúde mental em usuários da APS, contribuindo para a produção de conhecimento sobre a aplicabilidade da auriculoterapia no tratamento de ansiedade, com base em sua ação promotora de saúde mental, e subsídios para a formulação de políticas públicas e estratégias de ações, em busca da integralidade da atenção. O presente estudo possui como questão norteadora: quais os efeitos da auriculoterapia na promoção da saúde mental em usuários com queixa de ansiedade no contexto da APS?

## OBJETIVOS

Analisar os efeitos da auriculoterapia na saúde mental de indivíduos com ansiedade em seguimento na APS.

## MÉTODOS

### Aspectos éticos

Este estudo foi previamente submetido e aprovado pelo Comitê de Ética em Pesquisa da Escola de Enfermagem de Ribeirão Preto. Todos os participantes foram esclarecidos sobre as etapas e procedimentos do estudo e assinaram o Termo de Consentimento Livre e Esclarecido.

### Desenho, período e local do estudo

Foi conduzido estudo misto de caráter explanatório sequencial, com uma primeira fase de análise quantitativa, seguido por uma segunda fase de análise qualitativa^(15)^. A primeira fase (quantitativa) abrangeu o período de junho de 2021 a fevereiro de 2022, enquanto a segunda fase (qualitativa), de abril a junho de 2022, seguindo as orientações do *guideline COnsolidated criteria for REporting Qualitative research*.

Este estudo foi conduzido na Unidade Matricial de Saúde Valdemar Hial Júnior, localizada no município de Uberaba, Minas Gerais, onde são realizados aproximadamente nove mil atendimentos mensais, e conta com três equipes da Estratégia Saúde da Família (ESF), que são Santa Terezinha, Jardim Espírito Santo e Fabrício.

### População: critérios de inclusão e exclusão

Foram incluídos participantes, de ambos os sexos, com idade maior que 18 anos, atendidos na unidade de saúde com queixa de ansiedade na ficha de atendimento da unidade de saúde, classificados com nível de ansiedade variando de leve a extremamente severo na avaliação inicial do instrumento DASS-21^(16)^ e que não estavam realizando outra PICS. Foram excluídos indivíduos que apresentassem sinais de infecção, inflamação ou ferimento no pavilhão auricular, faziam uso de *piercing* na orelha (salvo brinco), estavam em período gestacional, estavam em surto psicótico ou fase aguda de alguma doença, não compareceram em duas ou mais sessões consecutivas, iniciaram tratamento medicamentoso para ansiedade, ou apresentaram diagnóstico de COVID-19 em um período menor que 15 dias do início do tratamento do estudo. Destaca-se que nenhum participante foi excluído na triagem inicial, visto que todos os usuários indicados pelos profissionais de saúde atenderam aos critérios de elegibilidade estabelecidos.

Para o presente estudo, adotou-se uma amostra de conveniência. Inicialmente, na fase quantitativa, a equipe de pesquisa reuniu-se com os profissionais de saúde das três equipes da ESF (Santa Terezinha, Jardim Espírito Santo e Fabrício). Durante a reunião, foram discutidos as indicações terapêuticas da auriculoterapia, suas potencialidades para o cuidado dos usuários e os objetivos da pesquisa. Os profissionais foram então solicitados a indicar usuários com ansiedade. Após a reunião, os profissionais forneceram inicialmente uma lista com 31 usuários. Cada um deles foi contatado por telefone e/ou pessoalmente e convidado a participar do estudo. Para ser incluído, o usuário deveria apresentar, na avaliação inicial do DASS-21, um nível de ansiedade variando de leve a extremamente severo. Esse critério foi adotado para poder avaliar o efeito da auriculoterapia sobre os escores de ansiedade do DASS-21 obtidos antes e após a terapia.

Para o cálculo do tamanho da amostra, utilizaram-se os recursos do *software* R *statistical package,* versão 4 (*R Foundation for Statistical Computing*, Viena, Áustria). O cálculo foi conduzido baseado nos escores de ansiedade do DASS-21, obtidos antes e após a auriculoterapia na amostra de 31 participantes inicialmente recrutada para o estudo piloto. Dessa forma, adotando a diferença dos 5 pontos nos escores de ansiedade do DASS-21, um nível de significância de 5% e poder do estudo de 90%. O tamanho amostral mínimo requerido foi de 60 participantes.

Os 31 participantes do estudo piloto foram incluídos na amostra final e, após nova conversa com a equipe, eles forneceram uma nova lista de 29 potenciais participantes. Eles foram contatados e aceitaram participar do estudo, mas dois desistiram por motivos pessoais, após completarem, respectivamente, sete e nove sessões de auriculoterapia. Para atingir a amostra de 60 participantes, foram adicionados dois novos usuários, que também aceitaram participar e que foram indicados pelos profissionais de saúde da unidade.

Na fase qualitativa, os participantes foram escolhidos intencionalmente com base nos resultados dos instrumentos de coleta de dados. Isso abrangeu os participantes que, após dez sessões de auriculoterapia, apresentaram níveis normais de ansiedade, percebendo uma boa qualidade de vida, satisfeitos com sua condição de saúde e aqueles que perceberam qualidade de vida ruim e insatisfação com sua condição de saúde. Nesta fase, aceitaram participar 17 participantes.

### Coleta de dados: instrumentos e procedimentos

Na fase quantitativa, realizou-se a coleta das características socioeconômicas dos participantes. Os escores de ansiedade, estresse e depressão foram avaliados pelo DASS-21^(16)^. Para a análise da qualidade de vida e satisfação dos participantes com sua saúde, utilizou-se o WHOQOL-Bref. Este instrumento engloba os seguintes domínios: físico, psicológico, relações sociais e meio ambiente^(17)^. Destaca-se que a aplicação da auriculoterapia na unidade de saúde pesquisada era realizada das 08:00 às 18:00, e cada participante tinha um horário marcado. Ao final das dez sessões de auriculoterapia, DASS-21 e WHOQOL-Bref eram reaplicados e analisados para avaliar a ansiedade, qualidade de vida e satisfação dos participantes com a sua saúde antes e depois da aplicação da auriculoterapia.

A fase qualitativa foi composta de entrevistas semiestruturadas, desenvolvidas e refinadas pelos pesquisadores, observação participante^(18)^ e diário de campo^(19)^, contendo registros das percepções dos participantes sobre as sessões de auriculoterapia e observações do pesquisador da realidade observada. Nessa etapa, os participantes foram contatados por telefone, e as entrevistas foram gravadas em celulares, ocorrendo individualmente em local privativo da Unidade Básica de Saúde. Com duração média de uma hora, os horários foram agendados conforme a disponibilidade dos participantes. As transcrições foram checadas duas vezes pelo pesquisador principal para garantir integridade, sendo posteriormente validadas pelos participantes via *WhatsApp^®^*.

Cada participante participou de dez sessões semanais de auriculoterapia, cada uma com duração de 30 minutos. Durante essas sessões, pontos específicos, como *Shenmen*, rim, sistema nervoso simpático, fígado, relaxamento muscular, tronco cerebral, coração, pulmão e ansiedade 1 e 2, foram estimulados usando agulhas descartáveis e estéreis de aço inoxidável de 0,18 x 8 mm. Após as sessões, as agulhas eram substituídas por sementes de vacária fixadas com fita microporosa antialérgica nos mesmos pontos auriculares. Os pontos auriculares eram alternados, e os participantes foram instruídos a estimular as sementes de vacária três vezes ao dia com digitopressão moderada, devendo removê-las após sete dias para evitar a saturação dos receptores nervosos e possíveis lesões por pressão. A definição dos pontos, sua localização e o número de sessões seguiram as evidências para ansiedade^(5,20)^. O pesquisador principal deste estudo, especializado em acupuntura, coletou os dados quantitativos e qualitativos, e seguiu as normas de biossegurança para aplicar a auriculoterapia (uso de luvas látex, algodão e álcool etílico 70% para higienizar o pavilhão auricular).

### Análise estatística

A análise de dados foi realizada utilizando os recursos do *software R statistical package*, versão 4 (*R Foundation for Statistical Computing*, Viena, Áustria). A análise descritiva incluiu as medidas resumo de frequência absoluta, relativa, média e desvio padrão. A análise comparativa da distribuição das categorias ordinais entre os tempos (antes e após) foi conduzida utilizando o teste de postos com sinais de Wilcoxon. O teste de Shapiro-Wilk foi utilizado para verificação do pressuposto de normalidade na distribuição das variáveis contínuas obtidas dos escores do DASS-21 e dos domínios do WHOQOL-Bref. Após este processamento, o teste t de Student para amostras dependentes foi aplicado para analisar as diferenças nos escores entre os tempos.

Além disso, modelos de regressão linear multivariados foram utilizados para o efeito da intervenção sobre os escores do DASS-21 e dos domínios do WHOQOL-Bref, ajustado para os fatores idade, sexo, estado civil, escolaridade e religião. Essa análise multivariada objetivou reduzir o viés de confusão na análise do efeito da intervenção e identificar outros fatores associados a mudanças nos escores. O Critério de Informação de Akaike^(21)^ foi utilizado para a seleção da variável dependente entre os modelos possíveis de contagem da classe GAMLSS, no caso do DASS-21, e entre o conjunto dos reais, no caso do WHOQOL-Bref. Para avaliar a adequação do modelo de regressão ajustado, foi aplicado sobre os resíduos do ajuste o teste de normalidade de Shapiro-Wilk. O nível de significância adotado para todos os testes foi de 5%.

### Análise qualitativa

Este processo foi alicerçado em uma tríade cíclica, envolvendo o referencial teórico^(12)^, entrevistas semiestruturadas e observação participante, e o uso do programa *ATLAS.ti*, versão 22, que auxiliou no processo de organização, codificação e otimização do processo analítico dos dados qualitativos^(22)^. Durante a análise de conteúdo temática^(18)^, seguiram-se as fases de pré-análise, exploração do material, tratamento e interpretação dos resultados obtidos. Durante a pré-análise, ocorreram a organização e a leitura flutuante do material, provenientes das entrevistas e da observação participante, que foram transcritas e inseridas no *ATLAS.ti.* Cada entrevista recebeu uma codificação por número sequencial dos participantes (P1, P2...). Na exploração do material, os temas foram agrupados conforme seus conteúdos, a partir das unidades de significado, até formar as categorias. Foi realizada a seleção/recorte das unidades das falas dos participantes (denominadas no programa citações/*quotations*), e criados e atribuídos códigos (*codes*), conforme as repetições de conteúdos semelhantes presentes em suas falas (citações), os quais foram agrupados em categorias (*families*). Na interpretação dos resultados, foram realizadas articulações entre citações, códigos e categorias, gerando redes de visualização, em forma de categorias temáticas, discutidas com base no referencial teórico sobre “APS como estratégia de organização do sistema de atenção à saúde”^(12)^.

## RESULTADOS

### Resultados da fase quantitativa

Um total de 60 participantes, 88,3% mulheres e 11,7% homens, com média de idade de 38,3 anos (amplitude de 18 a 66 anos), foram incluídos no presente estudo. A maioria relatou ser solteira (50%), com 48,3% possuindo o ensino superior completo. Entre os participantes com ensino superior, a maioria possuía curso superior, mas não atuava na área de formação e trabalhava em outra profissão, tais como auxiliar administrativo (n=06) e agente comunitário de saúde (n=3), sendo que um dos agentes comunitários era também profissional de educação física, maquiadora (n=1), autônomo (n=2), auxiliar de cirurgião dentista (n=1), representante comercial (n=1) e do lar (n=1). Havia também outros profissionais que estavam atuando na sua área de formação e que residiam no território, tais como enfermeiro (n=6), dentista (n=2), nutricionista (n=1), biomédica (n=1), veterinária (n=1), advogada (n=2) e serviço social (n=1). Observou-se que 35% referiram não pertencer a nenhuma religião. Quanto à profissão, a maior parte dos participantes era assistente administrava (13,3%).

Verificou-se uma redução do percentual das categorias de maior comprometimento de ansiedade, estresse e depressão na amostra depois do tratamento com auriculoterapia (p <0,001). Observou-se também melhoria estatisticamente significante no padrão de classificação da autopercepção de qualidade de vida e satisfação com a condição de saúde (p < 0,001). Destaca-se que, antes do tratamento, a maioria dos participantes (45%) avaliou a qualidade de vida como nem ruim nem boa, e 35% não estavam nem satisfeitos, nem insatisfeitos com a sua saúde. Após o tratamento 61,7% dos participantes passaram a avaliar a sua qualidade de vida como boa, e 46,7% ficaram satisfeitos com a sua saúde (Tabela 1).

**Tabela 1:** Classificação dos níveis de ansiedade, estresse depressão e qualidade de vida (WHOQOL-Bref) dos participantes antes e depois do tratamento da auriculoterapia (N=60), Uberaba, Minas Gerais, Brasil, 2023

Variáveis	Antes		Depois		Valor de *p*
	n	(%)	n	(%)	
Ansiedade					<0,001
Normal	0	(0,0%)	52	(86,7%)	
Leve	5	(8,3%)	3	(5,0%)	
Moderada	16	(26,7%)	5	(8,3%)	
Severa	2	(3,3%)	0	(0,0%)	
Extremamente severa	37	(61,7%)	0	(0,0%)	
Estresse					<0,001
Normal	2	(3,3%)	53	(88,3%)	
Leve	3	(5,0%)	5	(8,3%)	
Moderada	18	(30,0%)	2	(3,3%)	
Severa	22	(36,7%)	0	(0,0%)	
Extremamente severa	15	(25,0%)	0	(0,0%)	
Depressão					<0,001
Normal	11	(18,3%)	54	(90,0%)	
Leve	9	(15,0%)	2	(3,3%)	
Moderada	14	(23,3%)	4	(6,7%)	
Severa	12	(20,0%)	0	(0,0%)	
Extremamente severa	14	(23,3%)	0	(0,0%)	
Autopercepção da qualidade de vida					<0,001
Muito ruim	1	(1,7)	0	(0,0)	
Ruim	7	(11,7)	2	(3,3)	
Nem ruim, nem boa	27	(45,0)	11	(18,3)	
Boa	19	(31,7)	37	(61,7)	
Muito boa	6	(10,0)	10	(16,7)	
Satisfação com a saúde					<0,001
Muito insatisfeito	4	(6,7)	0	(0,0)	
Insatisfeito	17	(28,3)	7	(11,7)	
Nem satisfeito, nem insatisfeito	21	(35,0)	16	(26,7)	
Satisfeito	15	(25,0)	28	(46,7)	
Muito satisfeito	3	(5,0)	9	(15,0)	

*Nota: valor de p foi calculado utilizando o teste de postos com sinais de Wilcoxon.*

Houve reduções estatisticamente significantes após a intervenção para os valores dos escores de depressão, ansiedade e estresse observados no DASS-21 (p <0,001). O escore médio do nível de ansiedade era de 22,33 ±9,49 (antes) e 2,90 ± 3,30 (depois). Em relação ao estresse, antes o escore médio foi de 28,07 ± 7,30, e reduziu para 8,50 ±5,43. No escore de depressão, a média observada foi de 18,93 ±10,81 (antes) e 3,67 ±4,02 (após). Nos domínios da qualidade de vida dos participantes, houve um aumento estatisticamente significante (p <0,001) na média dos escores em todos os domínios do WHOQOL-Bref. Após o tratamento, o domínio físico apresentou média de escores mais alta (59,67 ±12,20), seguido pelos domínios de relações sociais (57,67 ±11,95), psicológico (55,44 ±9,99) e meio ambiente (54,88 ±11,77), presentes na Tabela 2.

**Tabela 2:** Medidas resumo dos escores dos instrumentos DASS-21 e domínios do WHOQOL-Bref dos participantes antes e depois do tratamento da auriculoterapia (N=60), Uberaba, Minas Gerais, Brasil, 2023

Variáveis	Antes		Após		Valor de *p*
	Média	Desvio padrão	Média	Desvio padrão	
Escores do DASS-21					
Depressão	18,93	10,81	3,67	4,02	<0,001
Ansiedade	22,33	9,49	2,90	3,30	<0,001
Estresse	28,07	7,30	8,50	5,42	<0,001
Domínios do WHOQOL-Bref					
Físico	41,29	13,25	59,67	12,20	<0,001
Psicológico	40,11	11,68	55,44	9,99	<0,001
Relações sociais	44,33	14,97	57,67	11,95	<0,001
Meio ambiente	46,79	11,46	54,88	11,77	<0,001

*Nota: valor de p foi calculado utilizando o teste t de Student para amostras dependentes.*

A Tabela 3 expressa a análise do modelo de regressão multivariado do efeito da intervenção sobre os escores de ansiedade, estresse e depressão, ajustado para idade, sexo, escolaridade, estado civil e religião. Os dados apontam para uma redução relativa média de 87,72% (Estimativa = -2,097; p <0,001) no domínio da ansiedade. O nível de escolaridade mais elevado levou à redução relativa média de 22,91% (Estimativa = -0,260; p = 0,019) no domínio da ansiedade. A intervenção apresentou redução relativa significante de 69,46% (Estimativa = - 1,186; p <0,001) na pontuação média do domínio do estresse. Houve redução relativa média de 82,51% (Estimativa = -1,743; p <0,001) na pontuação média da depressão. Nos participantes com escolaridade superior, houve redução relativa média de 37,02% (Estimativa = -0,462; p =0,001) na pontuação média da depressão. Sobre os domínios de qualidade de vida do WHOQOL-Bref, foram ajustados para fatores sociodemográficos.

**Tabela 3:** Modelo de regressão multivariado do efeito da intervenção sobre os domínios dos instrumentos DASS-21 e WHOQOL-Bref ajustado para fatores sociodemográficos, Uberaba, Minas Gerais, Brasil, 2023

Variáveis dependentes	Fatores	Estimativa ajustada	Erro padrão	t	Valor de *p*
Escores do DASS-21					
Ansiedade	Intercepto	2,355	0,282	8,336	0,000
	**Intervenção**	-2,097	0,167	12,499	0,000
	Idade	0,002	0,005	0,416	0,678
	Sexo masculino	-0,227	0,177	-1,283	0,201
	Estado civil (solteiro)	0,088	0,116	0,755	0,451
	Escolaridade	-0,260	0,110	-2,365	0,019
	Religião espírita	0,068	0,136	0,498	0,619
	Sem religião	0,130	0,137	0,948	0,344
	Religião outra	0,037	0,201	0,185	0,853
Estresse	Intercepto	2,696	0,172	15,637	0,000
	**Intervenção**	-1,186	0,091	-2,918	0,000
	Idade	-0,001	0,003	-0,605	0,546
	Sexo masculino	-0,116	0,102	-1,140	0,256
	Estado civil (solteiro)	0,018	0,070	0,267	0,789
	Escolaridade	-0,096	0,065	-1,467	0,145
	Religião espírita	0,034	0,084	0,411	0,681
	Sem religião	0,134	0,082	1,628	0,106
	Religião outra	0,060	0,117	0,510	0,610
Depressão	Intercepto	2,634	0,344	7,651	0,000
	**Intervenção**	-1,743	0,187	-9,324	0,000
	Idade	-0,004	0,006	-0,679	0,498
	Sexo masculino	-0,449	0,238	-1,883	0,062
	Estado civil (solteiro)	0,115	0,148	0,774	0,440
	Escolaridade	-0,462	0,138	-3,348	0,001
	Religião espírita	-0,149	0,170	-0,877	0,382
	Sem religião	0,018	0,166	0,110	0,912
	Religião outra	-0,118	0,260	-0,454	0,650
WHOQOL-Bref					
Físico	Intercepto	3,343	0,210	15,902	0,000
	**Intervenção**	0,981	0,094	10,433	0,000
	Idade	-0,011	0,005	-2,023	0,045
	Sexo masculino	0,353	0,161	2,193	0,030
	Solteiro	0,060	0,098	0,615	0,539
	Escolaridade	0,325	0,101	3,217	0,001
	Religião espírita	0,079	0,189	0,417	0,676
	Sem religião	-0,306	0,113	-2,712	0,007
	Religião outra	-0,198	0,288	-0,686	0,493
Meio ambiente	Intercepto	3,678	0,340	10,795	0,000
	**Intervenção**	0,387	0,098	3,958	0,000
	Idade	-0,014	0,005	-2,752	0,006
	Sexo masculino	0,242	0,144	1,678	0,096
	Solteiro	0,197	0,110	1,790	0,076
	Escolaridade	0,141	0,096	1,471	0,144
	Religião espírita	0,180	0,172	1,044	0,298
	Sem religião	-0,044	0,188	-0,234	0,814
	Religião outra	-0,076	0,217	-0,353	0,724
Psicológico	Intercepto	3,080	0,251	12,245	0,000
	**Intervenção**	0,766	0,088	8,707	0,000
	Idade	-0,003	0,004	-0,689	0,491
	Sexo masculino	0,339	0,145	2,338	0,021
	Solteiro	0,009	0,101	0,096	0,923
	Escolaridade	0,200	0,096	2,086	0,039
	Religião espírita	0,125	0,120	1,038	0,301
	Sem religião	-0,328	0,120	-2,733	0,007
	Religião outra	-0,192	0,168	-1,142	0,256
Relações sociais	Intercepto	1,303	0,077	16,91	0,000
	**Intervenção**	0,186	0,032	5,699	0,000
	Idade	-0,002	0,001	-2,227	0,027
	Sexo masculino	0,087	0,040	2,155	0,033
	Solteiro	0,007	0,026	0,291	0,771
	Escolaridade	-0,055	0,028	-1,960	0,052
	Religião espírita	0,034	0,034	0,979	0,329
	Sem religião	-0,061	0,038	-1,615	0,109
	Religião outra	0,007	0,048	0,155	0,876

O principal resultado da análise quantitativa foi que a intervenção por auriculoterapia apresentou efeito no aumento de todos os domínios de qualidade de vida. Além disso, uma idade maior estava relacionada a menores escores dos domínios físico, meio ambiente e relação sociais. O sexo masculino foi relacionado a maiores escores dos domínios físico, psicológico e relações sociais. A maior escolaridade foi relacionada a maiores escores dos domínios físico e psicológico. E a categoria sem religião foi relacionada a menores escores dos domínios físico e psicológico.

### Resultados da fase qualitativa

Na fase qualitativa, 17 participantes aceitaram participar. A maioria deles (n=14) era do sexo feminino, com idade média de 40,58 anos (26-58 anos). Além disso, 11 eram casados e seis eram solteiros, com a maior parte (n=8) possuindo nível superior completo, com predomínio de não pertencerem a religião (n=8) e agentes comunitários de saúde (n=3). A partir da análise de conteúdo temática dos dados qualitativos com o suporte do programa *ATLAS.ti*, versão 22, surgiram seis categorias (Quadro 1).

**Quadro 1:** Organização dos dados utilizando códigos (codes) e categorias (families), de acordo com a utilização do programa ATLAS. ti, versão 22, Uberaba, Minas Gerais, Brasil, 2023

Categorias (*familie*s)	Códigos (*codes*)
Conhecimento limitado dos usuários a respeito da auriculoterapia e de seu potencial de cuidado	Conhecimento da auriculoterapia; desconhecimento da auriculoterapia; pouco conhecimento da auriculoterapia.
Impacto da ansiedade na saúde física e mental	Agitação; agressividade; alergia na pele; ânsia de vômito; aumento da pressão arterial; aumento da vontade de comer; aumento da vontade de fumar; cansaço; conflitos com pessoas próximas; dificuldade para dormir; dor de cabeça; enjoo; falha na memória; impaciência; irritabilidade; nervosismo; pensamento acelerado; pensamentos negativos (morte e medo de algo ruim acontecer); preocupação; queda de cabelo; taquicardia.
Repercussões da pandemia nos fatores psicológicos, econômicos, sociais e físicos	Ansiedade; aumento da ansiedade por trabalhar em ambiente de saúde; convivência com perda de entes queridos; dificuldade financeira; dificuldade para dormir; distanciamento social; estresse; impacto na ocupação/trabalho; medo de morrer; medo de perder pessoas próximas; medo de ser infectado(a) pelo coronavírus; nervosismo; violência contra a mulher.
Benefícios da auriculoterapia para a promoção da saúde e mudança de estilo de vida	Autoconhecimento e calma para lidar com situações adversas; contribui para a qualidade de vida; contribui para a satisfação com a saúde; corresponsabilização no cuidado à saúde; equilíbrio da pressão arterial; manutenção da saúde; melhora da dor de cabeça; melhora da dor no corpo; melhora da tensão pré-menstrual (TPM); melhora do nervosismo e medo; melhora do relacionamento familiar; melhora do sono; melhora e controle da ansiedade; promove o despertar para um estilo de vida saudável; promove tempo para cuidar de si; redução da dosagem de medicamento para a ansiedade; redução da queda de cabelo.
Reconhecimento da auriculoterapia como recurso de cuidado em saúde significativo	Auriculoterapia como um recurso natural no cuidado à saúde; efeito da auriculoterapia é duradouro; recusa de tomar remédio alopático (dependência e efeito colateral).
Fatores negativos correlacionados à saúde mental, qualidade de vida e satisfação com a saúde	Conflito familiar; desorganização da rotina; dificuldade financeira; dor; enxaqueca; estilo de vida não saudável; exercício de múltiplos papéis; Fibromialgia; morte de um ente querido; relacionamento abusivo.

Na categoria 1, observou-se desconhecimento da auriculoterapia, sua oferta e potencial de cuidado na APS, conforme as falas:

*Já tinha ouvido falar. Mas, conhecimento eu não tinha.* (P5)

*Eu tinha, assim, muito superficial. Minha mãe fez esse tratamento, ela tinha problema de coluna, alguns problemas físicos, né?* (P39)

Na categoria 2, o impacto da ansiedade na saúde física e mental foram aspectos de maior número de códigos no *ATLAS. ti*. Sintomas físicos incluíram dor de cabeça, queda de cabelo, cansaço, alergia na pele, taquicardia, aumento da pressão arterial, enjoo e ânsia de vômito:

*Eu sentia tanto dor de cabeça. Era enxaqueca, era enjoo* [...] *a minha pressão estava muito alta* [...] *a minha queda de cabelo, absurda, muito forte.* (P57)

[...] *eu tive uma queda capilar também que eu cheguei a achar que eu iria ficar careca* [...] *eu estava tendo às vezes taquicardia, sabe? Em momentos de crise* [ansiedade]. (P2)

[...] *uma alergia* [na pele] *de ansiedade antes do tratamento* [...] *muita ansiedade, aí começava às vezes me dar ânsia.* (P41)

A ansiedade gerava pensamento acelerado, falha na memória, pensamentos negativos, preocupação, agitação, impaciência, irritabilidade, nervosismo e dificuldade para dormir:

[...] *eu estava sentindo um cansaço extremo, mas com agitação e pensamento bem acelerado e não dormia bem a noite* [...]. *E assim, eu fui ficando com problemas, com falhas na memória, raciocínio lento* [...]. (P21)

*Eu tinha uma mente muito acelerada* [...] *estava generalizada a minha ansiedade né, pensando que iria morrer a qualquer hora* [...]. (P55)

*Eu não dormia, eu não descansava, eu já acordava nervosa, eu já acordava acelerada* [...]. (P41)

Em decorrência da ansiedade, alguns participantes relataram recorrer à comida:

[...] *eu prejudicava o meu organismo porque eu descontava essa ansiedade na comida, em doce* [...] *eu ficava ansiosa, eu comia.* (P54)

[...] *não dormia direito, eu descontava a minha ansiedade em comer à noite.* (P57)

A ansiedade aumentava a vontade de fumar e ocasionava conflitos com as pessoas próximas:

*Não conseguia coordenar, ordenar os pensamentos, e isso acabava me desestabilizando mais ainda, deixando mais ansioso, fumando bastante, descontando, brigando com pessoas próximas a mim.* (P17)

Na categoria 3, observou-se que a pandemia de COVID-19 gerou ansiedade, preocupação, medo e morte de pessoas queridas:

*Minha ansiedade aumentou* [...] *eu perdi dois vizinhos meus, um de cima e o outro de baixo. Eu perdi dois primos* [...] *e aí começou a bater o desespero na gente e foi que eu fiquei meio desesperada* [...] *desesperei por causa da minha mãe também, por ser idosa. Eu, por ter pressão alta. A gente não podia pegar COVID.* (P57)

*Passei a ter crises de ansiedade* [...] *eu tinha muito medo de pegar COVID, muito medo de morrer. Muito medo de transmitir para minha família* [...] *eu fiquei muito tempo sem ir na casa delas* [...]. (enfermeira/P21)

*Eu comecei a ter essa ansiedade bem no início da pandemia* [...] *acho que pelo meu trabalho, por ser profissional de saúde né. A gente trabalhando na linha de frente.* (agente comunitária/P6)

Ademais, a pandemia ocasionou distanciamento social, dificuldades financeiras, e houve caso de violência contra a mulher:

*E também o isolamento, né? A gente teve que, era trabalho e casa, né? Você não tinha uma vida social mais.* (P6)

*No aspecto financeiro, porque como eu trabalho com eventos, eu me vi numa posição que eu não pude trabalhar mais* [...] *isso mexeu muito, muito com a minha vida econômica e psicológica* [...]. (P24)

*No começo da pandemia, eu passei por um trauma muito grande e isso me fez desenvolver. A ansiedade que eu já tinha ficou pior* [...] *ele* [ex-companheiro] *tentou tirar minha vida* [...] *eu consegui fugir* [...]. (P54)

Na categoria 4, observaram-se benefícios da auriculoterapia para a promoção da saúde, mudança de estilo de vida e no relacionamento familiar:

*Eu percebi que melhorou muito a ansiedade* [...] *melhorou, posso te dizer que melhorou 100%* [...] *melhorou muito mesmo, porque eu senti bem mais controle e relaxada.* (P5)

*O tratamento* [de auriculoterapia] *me deu uma desacelerada* [...] *agora ela* [ansiedade] *está estável, né? Eu consigo controlar* [...]. (P41)

*Ajudou muito, porque como é época pandêmica que a gente está em perigo. Até mesmo os problemas familiares* [...] *muda totalmente a postura que você passa a ficar mais calma.* (P29)

*Nossa, eu acho que eu sou outra pessoa. Eu estou super de boa* [...] *muito mais calma, dentro de casa com o meu marido, com meus filhos e com meu trabalho.* (P57)

O tratamento contribuiu para os participantes se corresponsabilizarem pela saúde e a ter um estilo de vida saudável:

*Estava uma pessoa sem fazer uma atividade, sem nada. Agora no momento, eu estou fazendo atividade; modificou bastante.* (P5)

*Emagreci cinco quilos porque comecei a controlar mais* [ansiedade], *diminuí a quantidade de alimentos, de coisa que eu estava comendo, de comer besteira.* (P17)

[...] *eu voltei a fazer exercício, então eu mudei completamente minha alimentação, não estou comendo açúcar* [...] *eu estou optando mais por fruta e verdura.* (P41)

Na categoria 5, alguns participantes expressaram preocupação com os efeitos colaterais dos remédios alopáticos e reconheceram a auriculoterapia como um recurso terapêutico não farmacológico que pode contribuir para o seu equilíbrio físico, mental, bem como para a promoção de sua saúde:

*Passei por psiquiatra. Ele tinha passado medicação forte pra mim, mas eu optei em não tomar. Eu tomei três dias e desisti* [...] e*u imaginei que fosse dar* certo [tratamento da auriculoterapia], *mas nem tanto igual foi. Eu esperava um resultado bem menor e foi além do que eu imaginava.* (P29)

*Eu não queria tomar remédio* [...] *acho que o tratamento* [da auriculoterapia] *não trata só a mente, ele trata, igual eu te falei, a mente, trata tudo assim. Eu acho que ele é melhor que o remédio, porque o remédio é momentâneo.* (P57)

Na categoria 6, observou-se que alguns participantes passaram pela experiência de perder membros da sua família, ocasionando prejuízos na saúde física, mental, além de na qualidade de vida e satisfação com a sua saúde:

[...] *mas equalizou foi na perda da família repentina* [...] e*ssa perda foi um infarto que o meu tio teve, né. Ele enfartou e eu passei mal, minha pressão subiu que eu fiquei assustado com isso e depois disso eu fiquei com medo, achando que iria acontecer comigo* [...]. (P55)

*Começou com a perda do meu pai* [...] *depois que ele faleceu, eu senti muita dor na coluna. Eu achei que eu estava com hérnia de disco. Fui no ortopedista* [...] *ele falou realmente, você está com fibromialgia.* (P24)

Alguns participantes não observaram melhora na qualidade de vida e satisfação com a sua saúde. Entre os motivos, destacam-se a multiplicidade de papéis, dificuldade financeira e conflito familiar:

[...] *eu tinha que mudar algumas coisas que eu estava, assim, fazendo ou permitindo que fizessem comigo e que precisava tomar decisões nesse sentido, aprender a dizer não, e dar prioridade, me priorizar em relação às outras pessoas* [...] *aprender a dividir as tarefas e não me sobrecarregar, um exemplo, em casa, né?* [...]. (P47)

[...] *chega financeiro. Familiar, tudo ruim. Um acompanha o outro, né? Por mais que a gente não queira. Uma coisa acompanha a outra e um estragando, quando um estragando já incomoda, agora imagina quando é tudo que estraga, é financeiro, é família.* (P4)

[...] *eu tenho muito problema em casa, especificamente com meu filho. Então, eu não consigo passar um dia bem.* (P24)

## DISCUSSÃO

Os resultados do presente estudo sugerem que a auriculoterapia pode contribuir com a redução dos níveis de ansiedade. Esses achados são corroborados por outros estudos^(1,23)^ que constataram efeitos positivos da auriculoterapia na ansiedade. A maior parte dos participantes com ansiedade pertencia ao sexo feminino. Tal dado está em conformidade com estudo prévio^(24)^, que indica que as mulheres podem ser mais acometidas por quadros de ansiedade em decorrência de diversos fatores, como maior pressão social e renda inferior em comparação aos homens, e muitas dessas mulheres adotam uma jornada de trabalho mais longa para conseguir manter as condições econômicas familiares.

Além da redução da ansiedade, também foi observada melhora nos sintomas de estresse e depressão, em consonância com os resultados de revisão sistemática^(5)^, que reforçou a eficácia da auriculoterapia na redução desses sintomas. Outro estudo^(25)^ também indica que a auriculoterapia pode ser útil para cuidar de pessoas que apresentam depressão.

Quanto à escolaridade dos participantes, a maioria possuía ensino superior completo. Esse dado contrasta com este estudo^(26)^, que revelou que grande parte dos usuários da atenção primária tinha ensino fundamental incompleto. Esse fenômeno pode ser explicado, pois houve relatos de participantes que estavam enfrentando dificuldades econômicas e ansiedade devido à pandemia de COVID-19, o que pode ter levado a aumento de indivíduos com nível superior a buscar tratamento na unidade de saúde em questão. A coleta de dados durante o período pandêmico pode ter contribuído para a mudança no perfil do público atendido pela unidade de saúde. Além disso, muitos participantes com diploma de ensino superior estavam atuando em áreas diferentes de sua formação original, como auxiliar administrativo, agente comunitário, maquiadora, autônomo, representante comercial, auxiliar de cirurgião dentista e do lar.

É importante destacar que o presente estudo iniciou durante o período da pandemia de COVID-19, fato este que pode ter impactado negativamente a saúde mental dos participantes, gerando ansiedade, medo do contágio e morte, afetando o convívio social e trabalho. Evidências têm mostrado que, além de desencadear uma crise econômica e sanitária, a pandemia de COVID-19 levou o Estado brasileiro a adotar o isolamento social como medida preventiva^(27,28)^. No entanto, um efeito colateral preocupante do isolamento social foi o aumento da violência doméstica contra a mulher, pois as vítimas passaram a ficar mais tempo em contato com o seu agressor, frequentemente sendo este o parceiro íntimo^(28)^.

Em relação à melhora da qualidade de vida e da satisfação com a saúde, os participantes apontaram que auriculoterapia possibilitou melhora da ansiedade, do sono, da dor de cabeça, redução da queda de cabelo e equilíbrio da pressão arterial. Ademais, muitos passaram a assumir um papel ativo na promoção de sua saúde, adotando um estilo de vida saudável que inclui a prática regular de atividade física e uma alimentação saudável. Tais achados corroboram os dados de estudos anteriores^(29,30)^ que apontam que as PICS podem aumentar a consciência do usuário da atenção primária sobre sua vida e sobre a importância de se autocuidar, além de contribuir para diminuir o uso de medicação e promover o protagonismo e empoderamento.

Observa-se que a incorporação das PICS na atenção primária pode contribuir não apenas para o estabelecimento de um SUS de qualidade, mas também para resolver os problemas de saúde dos usuários. Entretanto, de acordo com esses estudos^(5,31)^, as PICS são realizadas principalmente em ambientes terciários e secundários, o que reforça a necessidade da realização de mais estudos como este no âmbito da atenção primária, no intuito de fortalecer o reconhecimento dessas práticas, como o da auriculoterapia, enquanto estratégia de saúde pública no Brasil.

Dessa forma, é crucial que haja, no âmbito da atenção primária, educação permanente, para capacitar as equipes de saúde para o uso das práticas integrativas e divulgar a Política Nacional de Práticas Integrativas e Complementares para os profissionais de saúde e usuários da atenção primária, visto que muitos desses indivíduos desconhecem esta política e as potencialidades do uso dessas práticas não farmacológicas para a integralidade do cuidado em saúde^(30)^.

Destaca-se que, neste estudo, foi constatado que a auriculoterapia teve um impacto positivo na diminuição dos sintomas de ansiedade, resultando em uma melhoria na qualidade de vida e satisfação com a saúde dos participantes. No entanto, alguns enfrentaram desafios econômicos e sociais, como dificuldades financeiras, sobrecarga de responsabilidades e conflitos familiares, que afetaram negativamente sua qualidade de vida e satisfação com a saúde. Portanto, é crucial adotar uma abordagem interdisciplinar e intersetorial, envolvendo diferentes conhecimentos e setores da sociedade, para oferecer suporte àqueles que continuam a experimentar uma qualidade de vida e satisfação com a saúde insatisfatórias, mesmo após o tratamento com auriculoterapia. Nessa perspectiva, a intersetorialidade é uma estratégia eficaz para abordar os problemas de saúde agravados por determinantes sociais do processo de saúde-doença^(32)^, e o setor da saúde, especialmente a APS, deve atuar com outros setores e agentes para assegurar que as políticas públicas estejam alinhadas, a fim de maximizar seu potencial de contribuição para a saúde e o desenvolvimento humano^(12)^.

Uma das principais contribuições deste estudo reside na utilização do método misto explanatório, que permitiu a obtenção de resultados quantitativos e a sua explicação detalhada por meio de entrevistas com os participantes na fase qualitativa, sendo possível avaliar, de forma quantitativa e qualitativa, os efeitos da auriculoterapia na ansiedade, qualidade de vida e satisfação com a saúde. Os resultados podem ser de grande importância para as ciências da saúde, especialmente para as PICS. Os resultados desta pesquisa demonstraram que a auriculoterapia é uma terapia útil para reduzir a ansiedade, melhorar a qualidade de vida e a satisfação com a saúde, bem como para contribuir para a promoção da saúde mental e integralidade do cuidado. Essa descoberta pode ter uma contribuição significativa para a comunidade científica, que se dedica aos estudos sobre auriculoterapia, bem como para a formulação de programas, estratégias e políticas de saúde no âmbito da APS, ampliando as possibilidades terapêuticas para incrementar a integralidade da atenção à saúde e, assim, fortalecer o SUS.

### Limitação do estudo

Uma das limitações foi ter sido utilizado um único protocolo fechado de pontos de auriculoterapia. Assim, sugere-se o desenvolvimento de pesquisas futuras com pontos individualizados, seguindo os preceitos da MTC, com base no desequilíbrio energético, condição de saúde e necessidades específicas de forma não protocolar e individualizada que garante melhor potencial, porém o uso protocolar permitiu evidências e a disseminação da prática terapêutica através deste estudo. Também é importante considerar o possível efeito placebo em estudo que avalia efeitos terapêuticos. Dessa forma, sugerem-se estudos comparativos futuros que incluam outros tipos de grupos terapêuticos, com uso ou não de medicamentos, com o objetivo de investigar os efeitos da auriculoterapia em comparação a outros tipos de intervenção. Outra limitação foi a realização deste estudo durante a pandemia, o que pode ter diminuído o acesso e a amostra de participantes, o que afeta a generalização do estudo. Sugere-se o desenvolvimento de novos estudos longitudinais com seguimento mais longo para analisar os efeitos por um médio e longo prazo.

### Contribuições para a área da enfermagem

A enfermagem tem papel fundamental na qualidade de vida dos usuários na APS, e destaca-se atualmente no cenário de uso de PICS para assistência e pesquisa dentro do paradigma vitalista e da integralidade do cuidado. Nesse sentindo, é relevante enfatizar também a importância da atuação interdisciplinar e intersetorial, visando alcançar uma abordagem integral e holística no cuidado em saúde.

## CONCLUSÕES

Os achados do presente estudo sugerem que a auriculoterapia pode ser útil na redução dos níveis de ansiedade, estresse e depressão, e melhoria de aspectos relacionados à qualidade de vida e satisfação com a saúde dos participantes. Sua inserção na APS pode contribuir para fortalecer um sistema de saúde abrangente, que englobe prevenção, promoção e recuperação da saúde, com cuidado contínuo, humanizado e integral. É importante que os gestores de saúde capacitem os profissionais da APS para usar a auriculoterapia como parte do atendimento e para divulgar seus benefícios.

Recomenda-se a realização de mais estudos sobre a auriculoterapia para outras condições clínicas da APS, de forma a difundir e reconhecer essa abordagem terapêutica. Além disso, seria benéfico realizar estudos comparativos entre a seleção de pontos individualizados baseados na MTC e os pontos utilizados neste estudo, para uma análise mais detalhada dos efeitos da auriculoterapia na ansiedade, bem como a realização de estudos longitudinais para avaliar a eficácia da auriculoterapia.
